# Measures to address the stalled development of health law education in Chinese universities

**DOI:** 10.1186/s41256-022-00272-0

**Published:** 2022-10-24

**Authors:** Jingyi Xu, Yue Wang

**Affiliations:** 1 Georgetown University Law Center/O’Neil Institute for National and Global Health Law, 600 New Jersey Avenue, NW, Washington, DC 20001 USA; 2 School of Health Humanities, Peking University, No. 38 Xueyuan Road, Haidian District, Beijing, 100191 China

**Keywords:** Health law, Health education, Interdisciplinary subject, Systematic pedagogy, China

## Abstract

Health law education, an important part of global health education, is beneficial for both medical and law schools. This field can help lawyers and policy makers to develop their careers and equip traditional health professionals, such as doctors and nurses, with a basic knowledge of health law. However, unlike in western universities, health law education in China is still at its infant stage and, as such, lacks a systematic pedagogical approach among institutions of higher education in China. Considering the advancements in the field of health law education, this study systematically reviews the status of health law education in institutions of higher learning in China and suggests ways to make the pedagogical approach more consistent. This systematic review revealed that, between 2012 and 2021, major law schools and medical schools that have developed the subject of health law education in China lack consensus on the aim, scope, mode, and methods of health law education. The first problem is that Chinese universities are unable to agree on how to classify the subject of health law. Another set of problems relate to institutions themselves. Not only do universities lack qualified health law faculty, but they also rely on relatively uninspiring teaching materials. This leads to ineffective, generic pedagogical approaches in both medical and law schools. These problems leave future lawyers, future doctors and nurses unclear about their choices for health law study at the graduate level and their ultimate career development. We therefore propose four preliminary solutions to continue to develop this new interdisciplinary subject—health law education—in Chinese universities: clearly classify the subject of health law, equip the health law field with more professional textbooks, enact joint degree programs between medical schools and law schools, and establish a health law research center in either law schools or medical schools.

## Background

The ongoing worldwide pandemic of the coronavirus disease 2019 (COVID-19) highlights the significance of the field of health law education. Health law is a field of law with binding rules that govern the rights and responsibilities of a country’s government, health workers, companies, civil society, and population [1]. Together these rules make up the legal framework—or legal architecture—for health. The scope of health law, though, is quite broad (see Table 1). These rules can be used to create a formal commitment to fulfill the goal of universal health coverage, to solve a dispute between a hospital and patients, to regulate the safety of food and drugs, and to guide the work of health ministries, pharmaceutical companies, and civil society using mandates and policies [2].

**Table 1:** The scope of health law

Healthcare law	Health insurance law
	Laws regarding healthcare providers (including public and private hospitals, clinics)
	Laws regarding mental health issues (including depression, suicide, smoking and alcoholism)
	Laws related to the protection of women’s and children’s rights
Public health law	Establishment and reform of the public health system
	Food and drug law
	Laws regarding the regulation of tobacco sales, marketing, and use
	Laws regarding health technology
	Laws regarding infectious diseases and noncommunicable diseases
Global health law	Laws regarding global health security
	International Health Regulations (2005)
	The influence of international trade law on global health (represented by Doha Declaration)
	International human rights law (represented by International Covenant on Economic, Social and Cultural Rights)

As a new interdisciplinary subject, the international society has widely acknowledged that health law education has the potential to directly influence the future of health professionals and the future of a national health system both during the COVID-19 pandemic and in a post-pandemic world.

From the domestic view, health law has been a priority under the Healthy China 2030 Plan [3] and the 14th Five-Year Plan [4]. In addition, health law education can have a promising future since it has been identified as one of the new interdisciplinary disciplines, which is a development focus of the current higher education industry in China. In November 2021, the Academic Degrees Committee of the State Council issued Interdisciplinary Setting and Management Measures (Trial) [5]. In December 2021, President Xi Jinping proposed that the Chinese higher education industry should accelerate the development of interdisciplinary disciplines that have important practical significance at the 23^rd^ meeting of the Central Government Reform Committee [6]. Since then, interdisciplinary disciplines have been formally incorporated into the national education curriculum, and institutions are now required to provide additional information, ranging from the number of degrees awarded to students to the evaluation of academic achievements for faculty members.

From the international view, the benefits of legal education in health education have long been recognized [7]. For instance, when it comes to the graduates from medical schools, Shah argued that graduates from medical school should have an understanding of how the legal system works and how to navigate it. Otherwise, the practicing physicians may not be satisfied about their jobs due to the confusion about malpractice law, and they could be hopeless towards the complicated procedures from health insurance companies [8]. As for the healthcare lawyers, they need to get good command of health law since they often provide legal advice on informed consent, physician recruitment, patient confidentiality, among other health-related issues [9]. Gostin and his colleagues further pointed out the importance of the legal determinants of health, and how to harness the power of law for global health and sustainable development [10].

Despite the awareness of the increasing significance of health law education, the development of health law and health law education in China is just at the initial stage [11]. China does not currently have a systematic education plan for health law in Chinese universities, and, therefore, health law education has several issues.

Considering the large number of universities in China, it is not feasible to analyze the status of health law education in all the Chinese universities. We focused mainly instead on representative law schools and medical schools that have developed health law education for more than three years. To ensure the objectivity and generalizability of the research, we searched on the official websites of these universities to find all the relevant information about health law education. In addition, we used the name of the institutions and relevant keywords, such as “health,” “healthcare,” “medical,” and “law,” in the search engine to ensure the integrity of the study.

In this article, we identify problems in health law education and make suggestions about the following issues: the subject of health law is not clearly classified, the curricula are outdated, the pedagogical materials are a bit uninteresting, the faculty members do not have enough and specific health law education background, and the education is not quite tailored to the student population.

## The problems with health law education

### Not clearly classified subject

Chinese universities have long debated how to classify the subject of health law. From our analysis, the responses to this debate can be roughly divided into three categories. The first category is that, as a brand-new interdisciplinary subject in China, health law should be classified as an independent discipline [12]. Tsinghua University [13] and East China University of Political Science and Law [14] are two representative institutions that support this argument. The second category is that health law should be made a branch of administrative law [15]. This viewpoint takes into consideration that, at present, health law in China mainly includes food and drug law, prevention and surveillance systems of infectious diseases, and the national and local healthcare system. For instance, China University of Political Science and Law puts its Health Law Research Center under the leadership of School of Law-Based Government and under the supervision from College of Comparative Law [16]. The third category is that health law should be included in civil law [17]. This argument is based on the argument that most medical malpractice cases fall under tort law, which is a sub-category of civil law. For example, Southwest University of Political Science and Law [18] and Fudan University has classified health law as a subtopic of civil law [19]. It is clear that universities have no unified way of classifying the subject of health law. This unclear classification of health law not only creates disorder in the evaluation mechanism used to assess academic achievement, but it also allows faculty members to treat health law as a “fringe” subject that is not a central part of a law school education and, therefore, give it little attention.

### Outdated curricula and uninteresting teaching materials

Most Chinese universities that launch a health law curriculum pay much more attention to theoretical study than practical courses. This focus on theoretical study is somewhat problematic. Most health-related laws in China, a civil law country, are statutory, and Chinese students find these theoretical courses to be not that interesting [20]. On the other hand, health law education in the U.S. or Europe often offers stimulating practicum courses as a crucial part of its health law curriculum. For example, Georgetown University Law Center has a well-known health law major in western countries, and it offers courses like “Reproductive Health and International Human Rights Law” (a Project-Based Practicum) [21] and “Regulating Alcohol, Tobacco & Food in International and Comparative Law” (a Project-Based Practicum) [21]. The advantages of such practicum courses are that students have an opportunity to participate in actual work with external partners of the university (e.g., civil society organizations, pharmaceutical industries, and health departments from different governments), and the students can transfer what they learn from “paper” into “practice.” This curriculum tends to engage students as they can do health-related internships beside their classroom learning. This hands-on experience is especially beneficial for this student population because health law, a new interdisciplinary area with a very practical focus, requires students to accumulate enough experience to understand it [22].

In addition to the outdated pedagogical approach to the health law curriculum, the teaching materials for health law study in China are still not quite effective. Students have limited opportunities to learn about concrete cases in the health area, and what they face in their daily study is dull statutes that detail the health-related laws in China [23]. The lack of effective teaching materials in health law further makes the existing few teaching materials unengaging to students.

### Lack of qualified faculty members

The lack of faculty members with health law education background has hindered the development of health law education in China. Since health law is a relatively new subject in China, most current faculty members teaching this subject have a relatively limited background in the topic. The current health law faculty members in China have typically majored in other areas for their doctoral studies; they are either law professors specializing in administrative or civil law or medical school professors specializing in public health. An issue with the approach of these non-specialists is that they tend to choose a very specific area that fits their academic interests, and the students just learn the “tip of the iceberg” about health law [24]. In contrast, faculty members that teach health law at American universities often have a more focused educational background with health law as their one and only academic focus [25]. Professors with a high level of expertise tend to teach health law in a systematic and comprehensive way like other legal disciplines.

### Ineffective approach to health law programs in medical schools and law schools

Since health law is an interdisciplinary subject, both law schools and medical schools—especially public health departments and health humanities departments—have developed health law education in China. While this educational approach appears to be a promising way to promote health law in academia, the outcomes it creates are not ideal

Various medical schools, such as Southwest Medical University [26] and Dalian Medical University [27] have set up and conferred law degrees to full-time health law related majors at the undergraduate level. While other medical universities do not enroll students majoring in health law, most of them offer related courses in health law for medical school students. It can be problematic to offer a health law major for undergraduate level study because health law, as an interdisciplinary subject, requires expertise in two areas—law and health. This educational model has the potential to deprive students of a solid foundation in law or health, which would be a major challenge when pursuing their career path [28].

In law schools, health law majors are mainly offered at the postgraduate level. For example, Peking University [29], Tsinghua University [13], Fudan University [19], China University of Political Science and Law [16], East China University of Political Science and Law [14], Southwest University of Political Science and Law [18], and Jilin University [30] have all set up health law majors at the graduate level. This approach to designing health law programs is more reasonable than creating a health law major at the undergraduate level. In fact, most American and European countries also have a postgraduate set up [31]. However, the problem with Chinese law school is that students study law at the undergraduate level and know very little about the health or medical field. These students tend to feel at sea due to the lack of practicum courses and health law faculty members, and they often fail to understand what they are learning or have a clear idea about what they should do after graduation [32].

This model for health law programs, in which students major in health law at the undergraduate level in medical schools or law schools, creates a not satisfying outcome. Students risk having an incomplete command of their health major and becoming not qualified in both their legal career and their health career. In the end, students that pursue this model are not competitive with law students who specialized in other legal areas for judgeships or students from medical school for jobs in pharmaceutical companies.

## Measures for addressing the problems with health law education in China

The first measure we suggest is to classify health law as an independent subject rather than a lesser sub-subject of an existing legal department. Recently, scholars from the Peking University Health Science Center, the Chinese Academy of Medical Sciences and Peking Union Medical College Hospital have actively called for making “health humanities” first-level discipline under the larger category of “interdisciplinary” [33]. When this proposal is approved, health law would have a much better chance to develop as an independent second-level discipline under the “health humanities.” Since, in China, civil law is a second-level discipline of law [11], in this sense, health law would be at the same “discipline subject” level as civil law and other traditional legal disciplines.

For the second measure, since health law education is at an early stage in China, we suggest that Chinese scholars translate more health law related books from developed countries like the U.S., which have numerous, high-quality health law textbooks to choose from (see Table 2). We also recommend that Chinese universities develop practicum courses for health law study to give students a better idea of what this new interdisciplinary subject is and why it is important to study it.

**Table 2:** Selected health law textbooks from Western countries

Textbooks	Authors/Editors	Publication house
Health Law: Cases, Materials and Problems	Brietta Clark, Erin Fuse Brown, Robert Gatter, Elizabeth McCuskey, Elizabeth Pendo	Abridged (American Casebook Series)
Global Health Law	Lawrence O. Gostin	Harvard University Press
Foundations of Global Health & Human Rights	Lawrence O.Gostin, Benjamin Mason Meier	Oxford University Press
The Global Health Crisis: Ethical Responsibilities	Thana Cristina De Campos	Cambridge University Press
Soft Law and Global Health Problems: Lessons from Response to HIV/AIDS, Malaria and Tuberculosis	Sharifah Sekalala	Cambridge University Press
Global Health Governance: International Law and Public Health	Obijiofor Aginam	University of Toronto Press
Public Health Law: Power, Duty, Restraint	Lawrence O. Gostin, Lindsay F. Wiley	University of California Press

Our third suggested measure would ensure that health law programs have enough qualified faculty members by creating joint degree programs between law schools and medical schools either within the same university or between different universities. A good model for Chinese universities is the joint Juris Doctor (J.D) [34]/Master of Public Health (M.P.H.) degree program offered by Georgetown University Law Center and Johns Hopkins Bloomberg School of Public Health [35]. This joint degree program, described below, allows students to gain expertise in both health and law. First, a J.D./M.P.H. student spends his or her first year at the Law Center, learning the standard curriculum as a full-time J.D. student. Then, the student completes the M.P.H. degree at the Johns Hopkins Bloomberg School of Public Health [36]. Finally, the student completes the last two years of the J.D. curriculum at the Law Center [35]. Under this kind of joint educational model, students not only can systematically study both law and public health, but they can also take full advantage of the resources that the two universities offer. Georgetown University Law Center has accomplished law faculty members and Johns Hopkins has a highly skilled cadre of medical faculty members [35, 36]. Given the benefits of the joint degree program, we suggest law schools and medical schools in China cooperate either within the same universities or between different universities to help train future qualified faculty members.

Our fourth suggested measure, which tailors the education program to the student needs, is for Chinese universities to establish a health law research center in either the law school or the medical school setting. One successful example is the O’Neil Institute for National and Global Health Law at Georgetown University Law Center [37]. The biggest strength of such an institute is it can serve as a central hub that coordinates and designs a well-tailored educational program for students both from law schools and medical schools. The afore-mentioned joint degree program between Georgetown University Law Center and Johns Hopkins University was initiated by the O’Neil Institute. In addition, the O’Neil Institute has a jointly developed master’s program with the World Health Organization (WHO) [38]. Under this program, students spend one semester at Georgetown University Law Center to gain theoretical knowledge and then a semester at the WHO to gain practical experience [38]. This kind of educational model helps students to broaden their horizons while transferring what they learn from textbooks into practice.

China has started to develop these kinds of research centers, but these efforts are still at a nascent stage. The Law School of Central South University and most recently, East China University of Political Science and Law have played a leading role. Early in 2012, the Law School of Central South University and Xiangya Hospital of Central South University jointly established the Health Law Research Center [39] and opened a WeChat Public account called “Frontiers of Health Law” to provide the latest academic news in the field of health law. In August 2021, East China University of Political Science and Law started granting an interdisciplinary master’s degree in health law [14]. This change was made under the guidance of Ministry of Education, which symbolized the first time for health law to be an independent interdisciplinary subject. This is a good start for a well-tailored health education program, but, compared with health law development in western countries, more efforts are needed to build these kinds of lasting and effective programs [40].

## The way forward for health law education in China’s universities

In a nutshell, health law is a new interdisciplinary subject that is integral to global health education. Developing health law education in China’s universities is a major priority during the COVID-19 pandemic and in a post-pandemic world. Compared to current literature, the main contribution of our study is that we systematically analyzed the four biggest challenges for health law education in China and put forward four corresponding suggestions. Attaching more significance to the current health education system and making structural reforms can help health law education in China prepare health policymakers to fully address health issues, train doctors and nurses to completely value humanistic health care, and help China truly realize the right to health for every citizen under Universal Health Coverage.

## Data Availability

Not applicable.

## Abbreviations

COVID-19: Coronavirus disease 2019
WHO: World Health Organization
